# Marine particle microbiomes during a spring diatom bloom contain active sulfate-reducing bacteria

**DOI:** 10.1093/femsec/fiae037

**Published:** 2024-03-15

**Authors:** Robin Siebers, Doreen Schultz, Mohamed S Farza, Anne Brauer, Daniela Zühlke, Pierre A Mücke, Fengqing Wang, Jörg Bernhardt, Hanno Teeling, Dörte Becher, Katharina Riedel, Inga V Kirstein, Karen H Wiltshire, Katharina J Hoff, Thomas Schweder, Tim Urich, Mia M Bengtsson

**Affiliations:** Institute of Microbiology, University of Greifswald, Greifswald, Germany; Institute of Microbiology, University of Greifswald, Greifswald, Germany; Institute of Microbiology, University of Greifswald, Greifswald, Germany; Institute of Microbiology, University of Greifswald, Greifswald, Germany; Institute of Microbiology, University of Greifswald, Greifswald, Germany; Institute of Microbiology, University of Greifswald, Greifswald, Germany; Max Planck Institute for Marine Microbiology, 28359 Bremen, Germany; Institute of Microbiology, University of Greifswald, Greifswald, Germany; Max Planck Institute for Marine Microbiology, 28359 Bremen, Germany; Institute of Microbiology, University of Greifswald, Greifswald, Germany; Institute of Microbiology, University of Greifswald, Greifswald, Germany; Institute of Marine Biotechnology, 17489 Greifswald, Germany; Alfred-Wegener-Institute Helmholtz Centre for Polar and Marine Research, Biologische Anstalt Helgoland, 27498 Helgoland, Germany; Alfred-Wegener-Institute Helmholtz Centre for Polar and Marine Research, Biologische Anstalt Helgoland, 27498 Helgoland, Germany; Institute of Mathematics and Computer Science, University of Greifswald, 17489 Greifswald, Germany; Institute of Marine Biotechnology, 17489 Greifswald, Germany; Institute of Pharmacy, University of Greifswald, 17489 Greifswald, Germany; Institute of Microbiology, University of Greifswald, Greifswald, Germany; Institute of Marine Biotechnology, 17489 Greifswald, Germany; Institute of Microbiology, University of Greifswald, Greifswald, Germany; Institute of Marine Biotechnology, 17489 Greifswald, Germany; Alfred-Wegener-Institute Helmholtz Centre for Polar and Marine Research, Biologische Anstalt Helgoland, 27498 Helgoland, Germany

**Keywords:** *Desulfobacterota*, diatoms, networks, North sea, phytoplankton

## Abstract

Phytoplankton blooms fuel marine food webs with labile dissolved carbon and also lead to the formation of particulate organic matter composed of living and dead algal cells. These particles contribute to carbon sequestration and are sites of intense algal-bacterial interactions, providing diverse niches for microbes to thrive. We analyzed 16S and 18S ribosomal RNA gene amplicon sequences obtained from 51 time points and metaproteomes from 3 time points during a spring phytoplankton bloom in a shallow location (6-10 m depth) in the North Sea. Particulate fractions larger than 10 µm diameter were collected at near daily intervals between early March and late May in 2018. Network analysis identified two major modules representing bacteria co-occurring with diatoms and with dinoflagellates, respectively. The diatom network module included known sulfate-reducing *Desulfobacterota* as well as potentially sulfur-oxidizing *Ectothiorhodospiraceae*. Metaproteome analyses confirmed presence of key enzymes involved in dissimilatory sulfate reduction, a process known to occur in sinking particles at greater depths and in sediments. Our results indicate the presence of sufficiently anoxic niches in the particle fraction of an active phytoplankton bloom to sustain sulfate reduction, and an important role of benthic-pelagic coupling for microbiomes in shallow environments. Our findings may have implications for the understanding of algal-bacterial interactions and carbon export during blooms in shallow-water coastal areas.

## Teaser

During an algal bloom in the North Sea, bacteria living alongside oxygen producing diatoms exploit anoxic niches to reduce sulfate.

## Introduction

Microalgae inject gigatons of organic carbon into coastal oceans every year (Field et al. 1998) and phytoplankton blooms represent primary productivity hotspots. It has been estimated that over 90% of algal-produced carbon is consumed by heterotrophic bacteria in the immediate vicinity of algal cells, the phycosphere, during typical bloom situations (Seymour et al. 2017). Some of these bacteria are directly associated with living algal cells, or with sinking aggregates of senescent or dead algae, and therefore play an important role in the biological carbon pump. Despite their importance in carbon sequestration (e.g. Bligh et al. 2022), vertical connectivity (Mestre et al. 2018), and their documented complexity (e.g. Reintjes et al. 2023), particle-associated (PA) bacterial communities are less well understood than their free-living counterparts. In fact, they are often overlooked due to the sometimes fragile nature of particles, in combination with the practice of pre-filtration to exclude larger organisms prior to molecular analyses (e.g. Simon et al. 2002, Thiele et al. 2015, Heins et al. 2021).

Aggregates composed of living algae are known for a diverse microbiome of aerobic, heterotrophic bacteria that degrade complex algal organic matter, such as polysaccharide-rich exudates (Enke et al. 2019, Reintjes et al. 2023), although anaerobic metabolism such as diazotrophy is also known to occur (Riemann et al. 2022). Within this microbiome, bacteria are selected by factors such as host physiology and genotype (Ahern et al. 2021), the surrounding environment (Barreto Filho et al. 2021) and via stochastic processes (Stock et al. 2022). It is methodologically challenging to study associations between algal and bacterial taxa during natural phytoplankton blooms by direct observations, due to the transient nature of these associations (Seymour et al. 2017, Heins et al. 2021), as well as innate complexities and rapid dynamics of algal and bacterial communities during bloom events (Teeling et al. 2016). High-resolution temporal co-occurrence analysis, in combination with measurement of microbial functional potential, offers an indirect way to infer algae-bacteria associations, which can facilitate generating hypotheses about specific interactions and their potential functional implications.

A defining feature of marine particles are the steep chemical and redox gradients that PA bacterial communities are exposed to, compared to free-living, planktonic bacteria (Ploug et al. 1997). These gradients have been studied in the context of bathypelagic sinking particles (i.e. marine snow), which harbor micro-niches enabling anaerobic metabolism, such as microbial sulfate reduction (Shanks and Reeder 1993, Bryukhanov et al. 2011, Bianchi et al. 2018). Direct microelectrode measurement of oxygen concentrations in marine particles formed in roller tanks have demonstrated the importance of particle size and sinking velocity (Ploug et al. 1997), surrounding oxygen concentration (Ploug and Bergkvist 2015), but also the species composition of diatom detritus making up the particles (Zetsche et al. 2020) for the formation of anaerobic niches. Further, elevated concentrations of sulfide, inside artificial marine snow and field-collected particles compared to surrounding water masses, indicates that sulfate reduction takes place within such niches (Shanks and Reeder 1993). Sulfate-reducing bacteria have also been detected in oxygenated surface waters in the Black Sea, complementing observations of sulfate reduction above 30 m depth in these waters (Bryukhanov et al. 2011). Using a modeling approach, Bianchi and colleagues predicted that anaerobic particle microenvironments enabling for example sulfate reduction may be more widespread in the global ocean than previously assumed (Bianchi et al. 2018). In the photic zone, anaerobic micro-niches have been less widely investigated and the prevalence of sulfate-reducing bacteria is uncertain in the proximity of oxygen-producing living algal cells.

Here, we investigated microbial community dynamics of PA bacterial and eukaryotic taxa during a spring phytoplankton bloom in the southern North Sea at the shallow-water long-term ecological research site Helgoland Roads (Wiltshire et al. 2010) in the year 2018. We aimed to identify PA bacterial taxa co-occurring with the major eukaryotic taxa (diatoms and dinoflagellates) during the bloom. We hypothesized that bacteria co-occurring with diatoms would be compositionally and functionally distinct from those co-occurring with dinoflagellates. Using 16S and 18S rRNA gene amplicon data from a well-resolved time series (near daily sampling) collected between early March and late May in 2018, we constructed co-occurrence networks focusing exclusively on bacteria-eukaryote co-occurrences in the particle fraction (larger than 10 µm). In addition, we addressed bacterial functional gene expression by analysis of metaproteomes from three selected time points during the bloom.

## Materials and methods

### Sampling and sample processing

A large volume of seawater (40 L—140 L) was sampled using a clean bucket from 1 m depth below the water surface as previously described (Teeling et al. 2012, Wang et al. 2024) from the research vessel Aade in the morning at near daily intervals between beginning of March and end of May in 2018 at the long term ecological research (LTER) site Helgoland Roads (50° 11.3′ N, 7° 54.0′ E; DEIMS.iD: https://deims.org/1e96ef9b-0915-4661-849f-b3a72f5aa9b1). The site is located near the small island of Helgoland in the south-eastern North Sea and has a water depth of 6-10 m depending on tide (Wiltshire et al. 2010). For chlorophyll *a* (chl *a*) analysis, sample filtration was carried out in a laboratory under dim light to avoid the loss of pigments during the filtration process. We used a combined method of Zapata et al. (2000) and Garrido et al. (2003) for chl *a* extraction and analysis. Pigments were separated via high-performance liquid chromatography (HPLC) (Waters 2695 Separation Module), and detected with a Waters 996 Photodiode Array Detector. Secchi depth was measured from the vessel on site. The abundance of “detritus” (non-identifiable matter) was estimated microscopically on a scale from 0–6, corresponding to “none” (0), “moderate” (3) and “massive” (6) levels. Publicly available wind data from the weather station on Helgoland were obtained via the website www.wetterkontor.de. Water level data, collected at the harbor on the island, were obtained from http://www.portal-tideelbe.de/. For 16S and 18S rRNA gene amplicon sequencing and metagenome analysis, plankton biomass from a 1 L seawater subsample was filtered using 10 µm pore size polycarbonate membrane filters (47 mm diameter, Millipore, Schwalbach, Germany) to separate PA microbes (>10 µm) from smaller size fractions (not analyzed in this study). At three selected time points during the bloom (Julian days 107, 128 and 144, representing early-, mid- and late bloom phases), a separate filtration was performed using larger (142 mm diameter, Millipore) 10 µm pore size polycarbonate membrane filters for metaproteomic analysis. In order to maximize biomass harvest while avoiding clogging, filtered volumes varied between 15 and 30.5 L per filter for metaproteomic analysis. The >10 µm particle fraction comprises everything larger than 10 µm, and can include living phytoplankton cells, phytoplankton aggregates, zooplankton, fecal pellets and resuspended material of benthic origin. For the purpose of this study, we refer to microbes as particle-associated (PA) if detected in this filter fraction without making assumptions about the nature of the particle.

### rRNA gene amplicon sequencing and analysis

Samples from 52 time points throughout the bloom were collected and analyzed. DNA was extracted from the filters using the Qiagen DNeasy Power soil Pro kit (Qiagen, Hilden, Germany) according to the manufacturer's instructions. Dislocation of microbial cells from the filters and mechanical lysis were achieved by bead beating in a FastPrep 24 5 G (MP Biomedicals, Irvine, CA, USA). DNA concentrations were measured at a Qubit 3.0 fluorometer (Invitrogen, Carlsbad, CA, USA). Extracted DNA was amplified with primer pairs targeting the V4 region of the 16S rRNA gene [515f: 5′-GTGYCAGCMGCCGCGGTAA-3′, 806r: 5′-GGACTACNVGGGTWTCTAAT-3′ (Walters et al. 2016)] and the V7 region of the 18S rRNA gene [F-1183mod: 5′-AATTTGACTCAACRCGGG-3′, R-1443mod: 5′-GRGCATCACAGACCTG-3′] (Ray et al. 2016) coupled to custom adaptor-barcode constructs. PCR amplification and Illumina MiSeq (Illumina, San Diego, CA, USA) library preparation and sequencing (V3 chemistry) were carried out by LGC Genomics (LGC Genomics, Berlin, Germany).

Sequence reads free of adaptor and primer sequence remains were processed using the DADA2 package (v1.2.0) in R (Callahan et al. 2016). In summary, forward and reverse Illumina MiSeq reads were truncated to 200 bp, filtered (maxEE = 2, truncQ = 2, minLen = 175), dereplicated and error rates were estimated using the maximum possible error estimate from the data as initial guess. Sample sequences were inferred, paired forward and reverse reads were merged and chimeric sequences were removed using the removeBimeraDenovo function. The resulting amplicon sequence variants (ASVs) were taxonomically classified using the Silva database (nr 99 v 138.1, Pruesse et al. 2007) for 16S rRNA and the PR2 database (version 4.13, minboot: 50, Guillou et al. 2013) for 18S rRNA sequences using the build-in RDP classifier. 16S rRNA gene amplicon reads classified as chloroplasts and mitochondria, as well as the 18S rRNA gene reads classified as *Metazoa* (zooplankton) were removed prior to downstream analyses (a single time point was excluded due to suspected contamination). The diatom genera *Nitzschia, Navicula* and *Cocconeis* were classified as primarily benthic based on local reference literature (Hustedt 1959). Correlation analysis was carried out on the relative abundance of these benthic diatom genera and *Delsulfobacterota* against water level, wind speed, and Secchi depth data using linear regression (function lm).

Co-occurrence networks were generated in R using Spearman rank correlation, as described previously (Bengtsson et al. 2017). Briefly, we excluded rare ASVs with a total abundance <100 reads (16S) and <500 reads (18S) across the whole dataset. Then, pairwise correlations between all remaining ASVs were calculated using the rcorr function (Hmisc R package), followed by P-value adjustment for multiple testing (function p.adjust) using the Benjamini-Hochberg method (Benjamini and Hochberg 1995). For the final network, we considered exclusively correlations between 18S and 16S ASVs, and with a correlation coefficient >0.7 and an adjusted p<0.01. The network was then plotted using the igraph R package (Csardi and Nepusz 2006, R Core Team 2023).

### Construction of a metagenome-based database for metaproteomics

Illumina sequencing was performed at the Max Planck Genome Centre Cologne on DNA extracted from both 3-10 µm- and >10 µm filter fractions from eight selected time points (19 March, 12 April, 17 April, 26 April, 08 May, 11 May, 22 May and 29 May 2018–Julian days 78, 102, 107, 116, 128, 131, 142 and 149), using the HiSeq 2500 platform and 2×150 bp chemistry. Filtration and trimming of the reads were done as described previously (Francis et al. 2021). Read quality for each sample was confirmed using FastQC v0.11.9 (Andrews 2014). Assembly was performed with MEGAHIT v1.2.9 (Li et al. 2015) (kmer length: 21). Contigs below 2.5 kbp were removed using anvi-script-reformat-fasta from within Anvi'o v6.2 (Eren et al. 2015). Genes were predicted using Prodigal v2.6.3 (Hyatt et al. 2010) as implemented in the Prokka v1.11 annotation pipeline (Seemann 2014). These genes sequences were subsequently combined with a list of common contaminants to generate a database for peptide-spectrum-matching (PSM) after elimination of redundant sequences (97% redundancy) with CD-Hit (Li and Godzik 2006, Fu et al. 2012).

### Metaproteomics

Proteins were extracted from biomass filters of the 17th of April, 8th of May and 24th of May 2018 (Julian days 107, 128, and 144) as described previously (Schultz et al. 2020, Schultz 2022) and analyzed with liquid chromatography-tandem mass spectrometry in triplicates. Briefly, proteins were extracted via bead-beating followed by acetone precipitation. The extracts were separated and fractionated by 1D SDS-PAGE and in-gel trypsin digested. After desalting and concentration of the peptides using C18 Millipore® ZipTip columns, the samples were measured with an Orbitrap Velos^TM^ mass spectrometer (ThermoFisher Scientific, Waltham, MA, USA). After conversion into mgf file format using MS convert in ProteoWizard (Palo Alto, CA, USA), spectra were matched against a metagenome-based database containing 14764755 entries. Mascot (Matrix Science, London, UK) and Scaffold (Proteome Software, Portland, OR, US) were used for peptide-spectrum-matching, protein identification and protein grouping. Instead of setting an FDR-threshold, identification of protein groups was based on number of peptide-matches with a minimum of two, a protein threshold of 99% and a peptide threshold of 95%. Identified protein groups were annotated via Prophane v6.2.3 (Schiebenhoefer et al. 2020), using the Uniprot-TrEMBL (as of September 2021) and NCBI nr (as of February 2022) databases for taxonomic and EggNOG v5.0.2 (Huerta-Cepas et al. 2019) and TIGRFAMs 15 (Haft et al. 2001) databases for functional annotation with Prophane default settings. Taxonomic information for *Desulfobacterota* proteins was manually confirmed against the most recent NCBI nr database (blastp, https://blast.ncbi.nlm.nih.gov, September 2023). TrEMBL taxonomic information for *Desulfobacterales* was manually curated and set to phylum level for better comparability with the Silva database. Relative abundances were calculated as Normalized Spectral Abundance Factor (NSAF) values using the quantification method “max_nsaf” integrated in Prophane. Briefly, SAF values were calculated by dividing exclusive unique spectrum counts for each protein group by protein length of the longest sequence in that protein group. For normalization, SAF values were then divided by the sum of all SAF values of the sample.

### Data visualization

Stacked bar plots for rRNA gene amplicon data and metaproteomics were created with R version 4.3.0 using the tidyverse package (Wickham et al. 2019) in combination with the svglite, polychrome, patchwork, glue and ggnested packages.

## Results

### Particle microbial community dynamics during the course of the bloom

**Figure 1.**
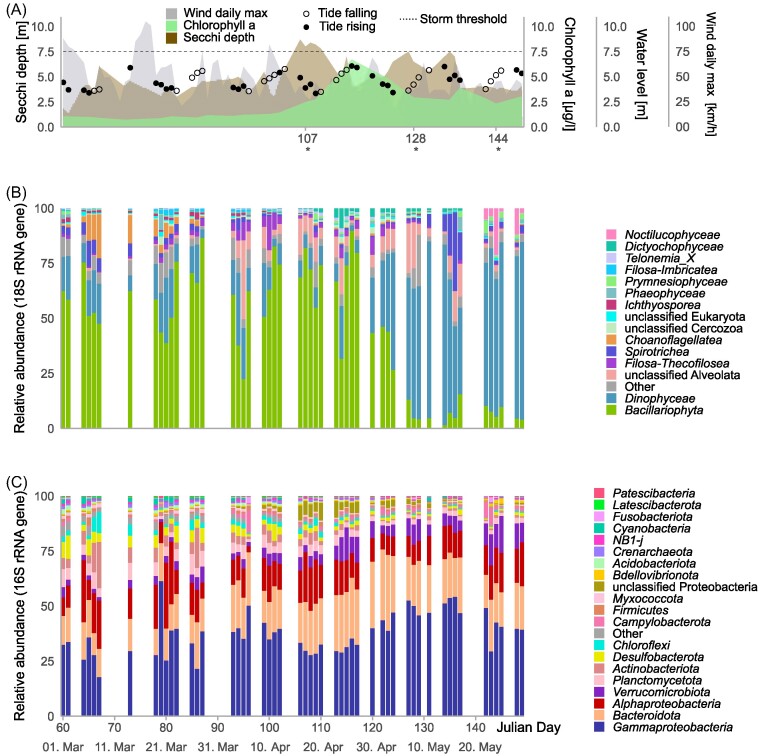
Progression of the 2018 spring bloom by Helgoland, North Sea. (A) Chl *a* (green) peaked around the 26th of April (Julian day 116), coinciding with a drop in Secchi depth (brown). Wind speed maxima (grey) reached storm levels (German: “Sturm”, >75 km/h, dotted line) on three occasions in the first half of the bloom. Points indicate water levels (m from global reference zero point) and tidal direction (white points: falling, black points: rising). Asterisks indicate time points (Julian days) for which metaproteomic sampling was performed. (B) 18S rRNA gene amplicon sequencing revealed that diatoms (*Bacillariophyta*) were the most abundant phytoplankton lineage, although dinoflagellates (*Dinophyceae*) dominated after the chl *a* peak. (C) 16S rRNA gene amplicon sequencing showed the highest relative abundances of *Desulfobacterota* (yellow) during the first half of the bloom.

The spring phytoplankton bloom in 2018 was characterized by an initial dominance of diatoms (*Bacillariophyceae*), followed by an increase in dinoflagellate (*Dinophyceae*) relative abundances after the bloom peak (peak in chl *a* concentration, Fig. 1A and B). The algal bloom (chl *a* measurements) peaked around the 26th of April (Julian day 116), coinciding with a decrease in water clarity, as indicated by Secchi depth rising at the onset of the bloom. 16S rRNA gene amplicon sequencing revealed a total of 17 615 ASVs in the particle bacterial microbiomes, which consisted mainly of *Proteobacteria (*e.g. *Spongibacteriaceae, Methylophagaceae, Rhodobacteriaceae), Bacteroidetes* (e.g. *Polaribacter*), *Verrucomicrobia* (e.g. *Persicirhabdus*) and *Planctomycetes* (e.g. *Phycisphaeraceae*). Further, *Actinobacteria* (e.g. *Illumatobacter*), and, notably, *Desulfobacterota* were also relatively abundant (Fig. 1C).

### Taxonomic and functional composition as detected by metaproteome analysis

**Figure 2.**
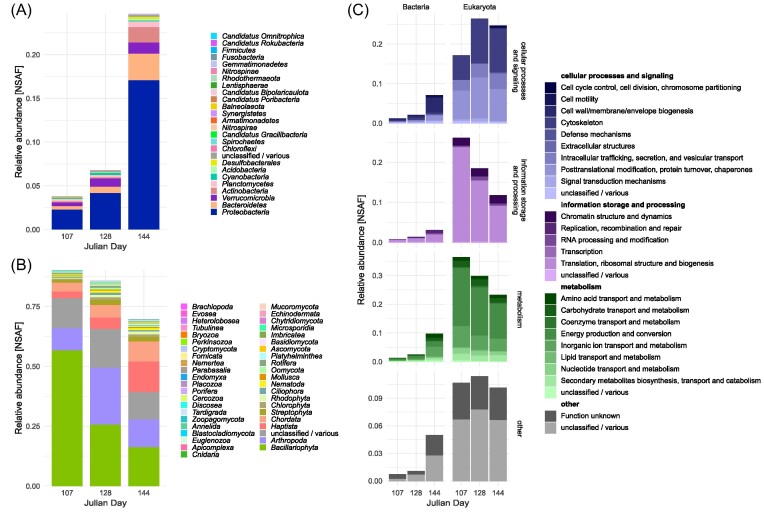
Taxonomic and functional annotation of particle metaproteomes from three selected time points during the bloom. (A) The proportion of bacterial proteins increased during the course of the bloom (B) Eukaryotic proteins made up the majority of identified proteins and showed an initial strong dominance of *Bacillariophyta* (C). Functional annotation of metaproteomes indicated a high contribution of metabolism-related eukaryotic proteins early in the bloom, while cellular processes and signaling increased during later time points.

Metaproteome sampling was performed on three time points that correspond to the early, mid and late phase of the bloom (Julian days 107, 128 and 144), as indicated in Fig. 1A). We detected 4 584 protein groups in >10 µm filter fractions from these sampled time points. Metaproteomic analyses confirmed high abundances of *Proteobacteria* and *Bacteroidetes* in the bacterial fraction (Fig. 2A), and dominance of *Bacillariopyhta* in the eukaryote fraction (Fig. 2B), with eukaryote proteins making up 90% of all proteins at the first selected time point. The proportion of eukaryotic proteins was reduced to 70% in the last selected time point (144), while bacterial proteins became comparably more abundant (25% on day 144 compared to 4% on day 107). Functional analysis revealed a predominance of eukaryotic proteins involved in metabolism, including energy production and conversion, and a shift towards expression of proteins relevant in cellular processes and signaling over the course of the bloom (Fig. 2C).

### Co-occurrence network analysis

**Figure 3.**
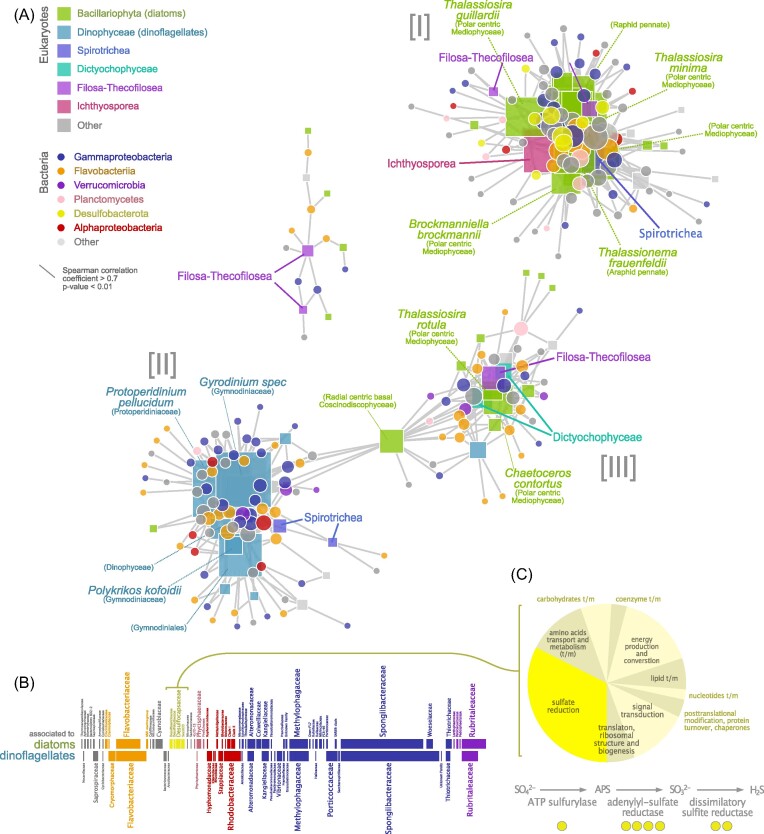
Co-occurrence of eukaryotes and bacteria, and detected protein groups of *Desulfobacterota*. (A) A network analysis of 18S rRNA (squares) and 16S rRNA (circles) gene ASV co-occurrences resulted in two major network modules containing diatom [I] and dinoflagellate [II] 18S ASVs, respectively, as well as one mixed module [III]. The network was calculated based on Spearman correlations (r >0.7, p < 0.01) exclusively between 18S ASVs and 16S ASVs. *Desulfobacterota* (yellow circles) were only associated with the diatom-dominated module I. The sizes of the symbols correspond to the number of significant correlations of the nodes (degree). (B) The bacterial taxa co-occurring with diatoms and dinoflagellates, respectively, are displayed as horizontal bars with a length proportional to the number of ASVs belonging to each lineage. *Desulfobacterota* (yellow) as well as potentially sulfur-oxidizing *Ectothiorodospiraceae* (*Gammaproteobacteria*, blue) were positively associated with diatoms but not with dinoflagellates. (C) The pie chart displays relative abundances for all 19 protein groups classified as belonging to *Desulfobacterota* during all three time points sampled for metaproteomics. Of these, seven protein groups (32%, bright yellow) represented enzymes involved in dissimilatory sulfate reduction. Yellow circles indicate which of the key enzymes in this metabolic pathway were detected.

Co-occurrence analysis resulted in distinct network modules centered around diatom- and dinoflagellate 18S rRNA gene amplicon sequence variants (ASVs). Based on significant positive correlations (Spearman Rho>0.7, corrected p>0.01) between eukaryotic 18S rRNA gene and prokaryotic 16S rRNA gene ASVs, including the 51 analyzed time points during the 2018 spring bloom, the network was dominated by three major distinct modules (Fig. 3A). Two of these modules were dominated by eukaryotic ASVs belonging to diatoms and dinoflagellates, respectively, while the third module mostly contained diatom ASVs and was linked to the dinoflagellate-dominated network module. Along the time line of the bloom, these modules roughly corresponded to the phytoplankton taxa prevalent in the early stages of the bloom (module I, mainly diatoms), during the late stages of the bloom (module II, mainly dinoflagellates) and mid-bloom around peak chl *a* (module III, Fig. 3A, Fig. 1A). The composition of bacterial ASVs that co-occurred with diatoms and dinoflagellates is depicted in Fig. 3B, and in more detail in supplementary Fig. 1. Eleven ASVs belonging to the *Desulfobacterota* were part of the main diatom-dominated network module I and co-occurred exclusively with diatoms, including the genus *Desulfosarcina*, and the families *Desulfocapsaceae* and *Desulfobulbaceae* as well as the lineage Sva1033 (Ravenschlag et al. 1999). In addition, 4 ASVs classified as *Ectothiorhodospiraceae* (genus *Thiogranum*) also co-occurred with diatoms (Fig. 3B).

### Analysis of *Desulfobacterota* proteins

Out of the 4 584 protein groups detected by metaproteome analysis, 19 were classified as belonging to different *Desulfobacterota* orders (Table S1). The relative abundance of predicted *Desulfobacterota* proteins averaged over all time points in the metaproteome was 0.14% (expressed as normalized spectral abundance factor—NSAF). This order of magnitude corresponded well to the relative abundance of *Desulfobacterota* ASVs in the microbiome at the same time points (average 0.38%, Fig 1C). Remarkably, no less than 32% of the *Desulfobacterota* metaproteome fraction (in terms of protein abundance) consisted of key enzymes for dissimilatory sulfate reduction (Fig. 3C). Of these, ATP sulfurylase, both the alpha and the beta subunits of dissimilatory sulfite reductase (DSR) and adenylyl-sulfate (adenosine-5′-phosphosulfate) reductase (APS reductase) were detected. We used a conservative threshold for protein identification of at least two matching peptides to consider a protein as validly detected.

### Assessment of sediment influence

**Figure 4.**
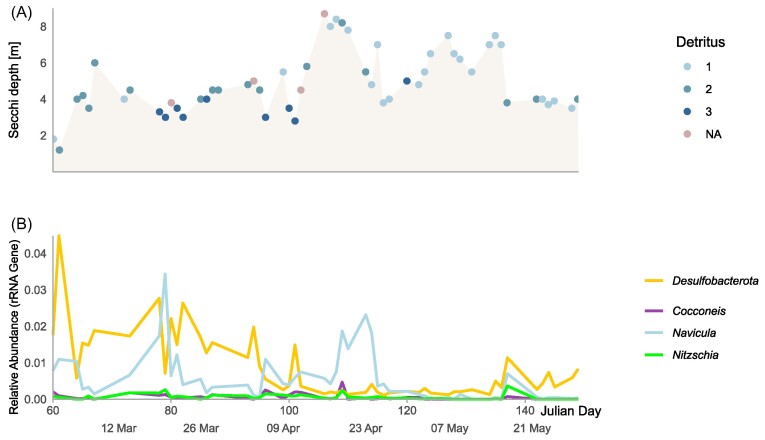
(A) Secchi depth and abundance of “detritus” (unidentifiable material) along the course of the bloom. Detritus levels were estimated under the microscope on a scale between 0 and 6 (0:“none”, 3: "moderate" 6:“massive”). (B) Relative abundance of *Desulfobacterota* (16S rRNA gene) in relation to relative abundances of known benthic diatom taxa (*Cocconeis, Navicula, Nitzschia*, 18S rRNA gene).

**Figure 5.**
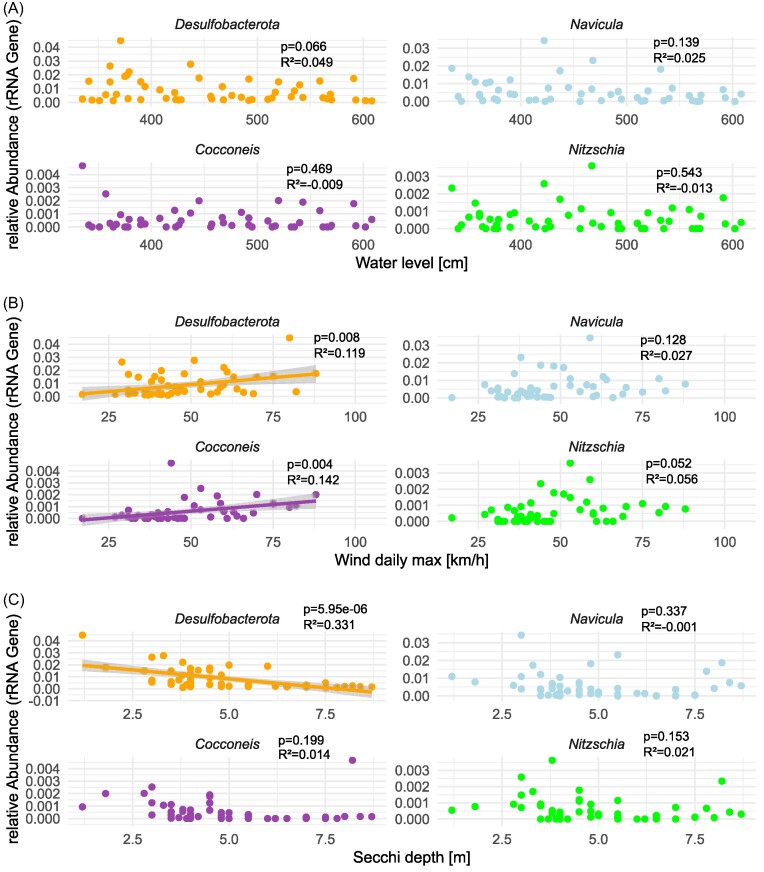
Correlations (linear regression) between *Desulfobacterota*, benthic diatom taxa (*Cocconeis, Navicula, Nitzschia*), and (A) water level, (B) wind speed, and (C) Secchi depth.

In order to assess the potential influence of resuspension of anoxic sediments on our results, we analyzed local wind speed maxima, tide levels and Secchi depth (Fig. 1A), as well as detritus levels, and the abundance of benthic diatom taxa (Fig. 4) along the course of the bloom. The relative abundance of total *Desulfobacterota* ASVs correlated significantly with Secchi depth (R^2^ = 0.33, *P* < 0.01) and with wind speed (R^2^=0.11, *P* < 0.01), but not with water level (R^2^ = 0.05, p>0.05), as illustrated in Fig. 5. In addition, low Secchi depth coincided with high estimated levels of “detritus”, i.e. microscopically unidentifiable particular material (can have both benthic and pelagic origin, Fig. 4A). The relative abundance of known benthic diatom genera was low (at most <0.05% of 18S amplicon reads, Fig. 4B). The benthic diatom genus *Cocconeis* significantly correlated with wind speed (R^2^=0.14, *P* < 0.01), indicating resuspension from benthic environments. Further, we searched for other known sediment-associated organisms, such as *Bathyarchaeota*, in the 16S amplicon dataset, which were not detected. We also did not detect any enzymes involved in denitrification (nrfA, narG, napA, nirK, nirS, nor and nosZ) in the metaproteomes.

## Discussion

The succession we observed during the 2018 spring bloom, with an initial dominance of diatoms, followed by dinoflagellates, using 18S rRNA gene amplicon sequencing agrees with the typical phytoplankton community succession at Helgoland Roads (Wiltshire et al. 2008, Käse et al. 2020). Metaproteome analysis highlighted the dominance of eukaryotic proteins in the sampled particles, making up 70–80% of detected proteins, most of which belonged to the major diatom phytoplankton. The high prevalence of proteins involved in basic metabolism such as energy production and conversion is consistent with the sampled >10 µm particle fraction mostly comprising of living phytoplankton cells, especially at the beginning of the bloom. However, bacterial proteins were present in all of the three selected time points, with a notable peak at day 144, and their taxonomic composition was very similar to that observed via 16S rRNA gene amplicon sequencing. Overall, the composition of particle bacterial microbiomes agreed with other reports from similar environments (Wang et al. 2024, Crump et al. 1999, Schultz et al. 2020, Heins et al. 2021, Reintjes et al. 2023).

As hypothesized, bacteria co-occurring with abundant diatoms formed a distinct network module, with taxa that were different from those co-occurring with dinoflagellates in a second distinct module. A third network module contained mostly diatom taxa, but also one dinoflagellate ASV and other phytoplankton taxa. These network patterns offer an alternative way to visualize the temporal dynamics of the bloom, and should not be interpreted as evidence of physical interactions between taxa (Röttjers and Faust 2018). However, one pattern that is striking in our network analysis is the exclusive co-occurrence of *Desulfobacterota* with diatoms.

We observed a high relative abundance of *Desulfobacterota* (in total 0.38% of bacterial amplicon reads) in particles (>10 µm), especially in the early phase of the bloom when diatoms were dominating. Despite the limited resolution of metaproteome data compared to DNA-based methods, desulfobacterial proteins were represented at all selected time points (in total 0.14% of metaproteomes). Network analysis further highlighted the temporal co-occurrence of *Desulfobacterota* with several diatom taxa. This raises the question of the niche filled by these anaerobic bacteria during an active phytoplankton bloom.

Diatoms, such as *Thallassiosira* spp. and *Thalassionema* spp., which were co-occurring with *Desulfobacterota* in this study (Fig. 3A), are known to form aggregates (Thornton 2002). For example, several species of *Thalassiosira* extrude long chitin fibrils, which prevent sinking and bind exopolymeric substances (EPS) also produced by the algal cells (Herth and Barthlott 1979, Den et al. 2023). This creates a favorable environment for bacteria to attach, which can in turn stimulate algal EPS production (Gärdes et al. 2011). EPS makes particles adhesive and thus bacteria can be captured in this sticky EPS layer. Smaller particles can aggregate to larger particles by collision and adhesion, in particular during phytoplankton blooms with high particle densities. Such aggregates can feature high numbers of living, photosynthesizing algal cells (Thornton 2002) which produce ample oxygen during the day, but at night respiration by the algal cells and their surrounding bacteria may deplete oxygen sufficiently for low oxygen or even anaerobic micro-niches to form. Experiments with marine particles in laboratory roller tanks have demonstrated how oxygen-depleted zones can form in diatom-derived particles (Ploug and Berggkvist 2015), in part due to EPS associated to the algae rendering the particles impermeable to water flow (Zetsche et al. 2020). Interestingly, sulfate reduction was detected in such particles in an earlier study, and the reducing microzones where this process was presumably taking place were frequently associated with diatom frustules (Shanks and Reeder 1993).

*Desulfobacterota* have not been identified as frequent members of diatom microbiomes in either cultures or in the field so far (Helliwell et al. 2022). However, a recent global survey of the diatom interactome detected positive correlations between diatoms and sulfate-reducing bacteria (*Desulfovibrio*) in samples from the Tara Oceans expedition (Vincent and Bowler 2020). In addition, *Desulfobacterota* have previously been repeatedly detected in particle-associated communities in the photic zone (Crump et al. 1999, Liu et al. 2020, Hallstrøm et al. 2022). In a parallel study, we reconstructed a genome of a *Desulfobacterota* member from metagenomic data from material sampled during the same phytoplankton bloom (Wang et al. 2024). *Ectothiorhodospiraceae* are purple sulfur bacteria (belonging to *Gammaproteobacteria*), that oxidize reduced sulfur compounds as electron donors during anoxygenic photosynthesis and are anaerobic to microaerophilic (Imhoff et al. 2022). They can oxidize H_2_S, e.g. produced via dissimilatory sulfate reduction by members of the *Desulfobacterota*. Our observation of *Desulfobacterota* as well as *Ectothiorhodospiraceae* co-occurring with diatoms is consistent with potential sulfur cycling during the diatom-dominated phase of this phytoplankton bloom under anoxic to very low oxygen conditions. While co-occurrence of diatom and *Desulfobacterota* rRNA genes does not by itself indicate that sulfate reduction is taking place in diatom-derived particles, detection of key functional enzymes by metaproteomics suggests that *Desulfobacterota* were actively carrying out sulfate reduction and thus gaining energy through anaerobic respiration during the bloom. The higher detection of these enzymes especially at the last time point (Julian day 144, Table S1 can likely be attributed to the larger proportion of bacterial proteins at this time point, when the diatom bloom was declining.

Temporal associations, such as detected by our co-occurrence network analyses, have to be interpreted with caution as additional variable factors that were not taken into account may influence the observed correlations. Importantly, we cannot quantitatively assess the influence of underlying sediment microbiomes at the rather shallow sampling site (6-10 m depth depending on tide), which frequently become resuspended during times of heavy wind thereby introducing anaerobic microbes to the pelagic environment. Indeed, some of our results point towards a significant influence of sediment microbiomes, such as the correlation between relative abundance of total *Desulfobacterota* ASVs and secchi depth, as well as *Desulfobacterota* and wind speed. The detected *Desulfobacterota* ASVs (e.g. classifying as *Desulfosarcina, Desulfocapsaceae, Desulfobulbaceae*) are related to typical benthic lineages (Ravenschlag et al. 1999), suggesting that they originated from the underlying sediments. The diatoms co-occurring with *Desulfobacteria* were primarily classified as common pelagic genera such as *Thalassiosira* and *Thalassionema*. However, the genus *Brockmanniella*, which can also inhabit benthic biofilms (Hernández Fariñas et al. 2017), was also found within network module I. The primarily benthic diatom genera *Nitzschia, Navicula* and *Cocconeis* were not part of the network, but *Cocconeis* relative abundances correlated with wind speed, indicating a turbulence-driven benthic-pelagic coupling. Nevertheless, a benthic origin of the *Desulfobacterota* detected in our study does not exclude physical interactions with the blooming pelagic diatoms. In shallow environments, benthic and pelagic microbiomes are in close contact within the photic zone, and seeding of pelagic particles by benthic microbes is likely frequent, which may explain the observed co-occurrences. In fact, most of the enzymes involved in sulfate reduction were detected in the last timepoint, when the diatom bloom was declining and wind speeds were moderate, indicating low sediment resuspension. Microscopic analysis revealed high abundances of the haptophyte phytoplankton *Phaeocystis globosa* were also detected at this timepoint (Wang et al. 2024), which may have contributed to aggregate formation (Schoemann et al. 2005)

Importantly, our methodology does not allow us to determine the physical proximity of *Desulfobacterota* and diatom cells. We therefore cannot rule out that the observed co-occurrences reflect a purely temporal association between pelagic diatoms and resuspended sediment bacteria. Indeed, demonstrating a potential physical association between *Desulfobacterota* and diatoms would require *in situ* microscopic investigation using for example taxon- or gene- specific fluorescent probes. With this approach, sulfate reducers were for example detected in oxygenated surface waters in the Black Sea (Bryukhanov et al. 2011). Likewise, it has yet to be clarified, whether any such temporal or physical associations are annually recurrent, under what specific conditions they occur, and which specific diatom taxa are involved. Thus, further studies are needed to confirm whether our results are representative for phytoplankton blooms in shallow water coastal areas.

Our results highlight the complexity of particle microbiomes and corroborate the need to study algae-bacteria particles as spatially heterogeneous entities. The microbiomes of aggregate-forming phytoplankton may indeed be similarly complex as those of animals and other multicellular organisms, featuring distinct micro-niches with sub-microbiomes (analogous to e.g. human skin vs. gut microbiomes), whose compositions depend on the chemical environment on a small spatial scale. Anaerobic micro-niches in phytoplankton-derived aggregates may affect carbon cycling insofar, as sulfate reduction can chemically alter particulate organic matter, rendering it resistant to microbial degradation (Raven et al. 2021). However, this effect has been demonstrated in oxygen-deficient zones of the ocean, and it is unclear this can be expected in oxygenated surface waters. Sulfate reduction in marine particles has been predicted to be prevalent in vast hypoxic (<60 µM oxygen concentration) waters of the global oceans, based on modeling of particle properties and oxygen regimes (Bianchi et al. 2018). The model could explain observed precipitation of Cadmium sulfide (CdS) in these waters (Janssen et al. 2014). Surface waters in the photic zone at our sampling site are expected to feature far higher oxygen concentrations at around 300 µM (7-10 mg/l, https://dashboard.awi.de/?dashboard=34404). However, anaerobic niches in marine phytoplankton-derived particles were already frequently reported in the context of nitrogen fixation (i.e. diazotrophy, e.g. Riemann et al. 2022). Recent work has shown that many *Desulfobacterota* are in fact capable of both diazotrophy and sulfate reduction (Liesirova et al. 2023) and that diazotrophic *Desulfobacterota* can move towards phytoplankton-derived organic matter via chemotaxis. Similar to diazotrophs, sulfate reducers in turn provide additional niches for bacteria which scavenge their metabolic products, i.e. sulfur oxidizers such as the observed *Ectothiorhodospiraceae*, leading to higher metabolic diversity and complexity within particles. We suggest that incorporation of sulfate reducing bacteria in phytoplankton-derived aggregates may be a relevant phenomenon, especially in shallow coastal areas where the microbiomes of underlying sediments provide a pool of abundant sulfate reducers to colonize aggregates. Considering the significance of marine particle microbiomes in carbon sequestration, it is vital to understand the consequences of their metabolic and compositional complexity, and the resulting microbial interactions in these microbiomes.

## Supplementary Material

fiae037_Supplemental_File

## Data Availability

Metagenome raw reads and assembled contigs used for metaproteome assignment are publicly available at the European Nucleotide Archive (ENA) under the project accession number PRJEB38290. 16S- and 18S-rRNA gene amplicon data are also available via ENA under the project accession number PRJEB51816. Mass spectrometry proteomics data have been deposited to the ProteomeXchange Consortium via the PRIDE (Perez-Riverol et al. 2022) repository with dataset identifier PXD035982.
