# Supplementary material for: Marine particle microbiomes during a spring diatom bloom contain active sulfate-reducing bacteria
Source: FEMS Microbiol Ecol. 2024 Mar 15;100(5):fiae037. doi: 10.1093/femsec/fiae037 (PMC11008741; doi:10.1093/femsec/fiae037)
Supplement: fiae037_Supplemental_File [file fiae037_supplemental_file.docx]

*Supplementary Material*

Marine particle microbiomes during a spring diatom bloom contain active sulfate reducing bacteria

[Supplementary figures 2](#__RefHeading___Toc570_4008211538)

[Supplementary tables 3](#__RefHeading___Toc572_4008211538)

# Supplementary figures


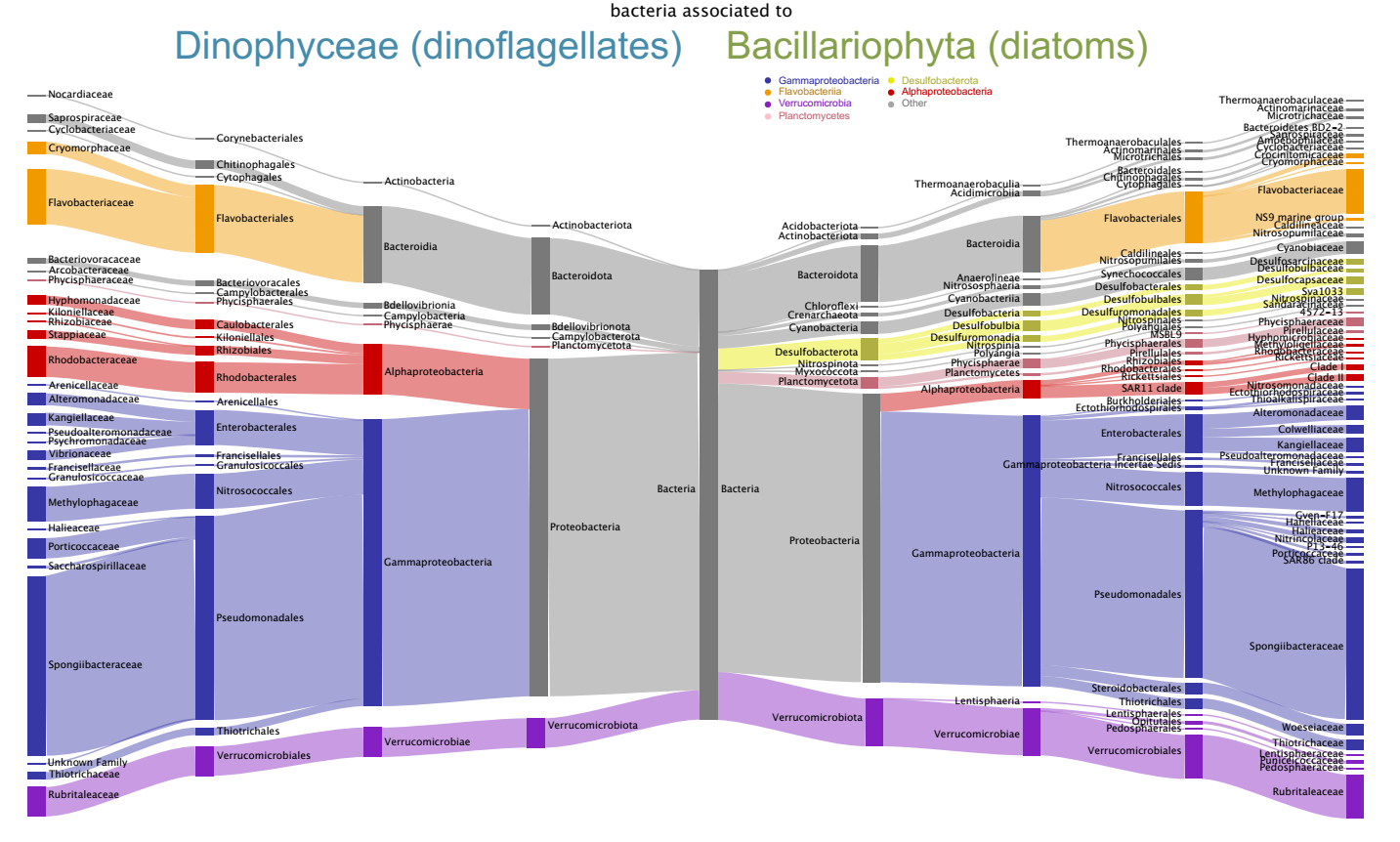


**Supplementary Figure 1:** Association of bacterial taxa (16S rRNA gene amplicon ASVs) with diatoms and dinoflagellates based on the co-occurrence network analysis. *Desulfobacterota* (yellow) and *Ectothiorodospiraceae* (blue) were only co-occurring significantly with diatoms.

# Supplementary tables

**Supplementary Table 1:** Seven key enzymes associated with the sulfur cycle detected with metaproteomics. ATP sulfurylase, adenylylsulfate-reductase and dissimilatory sulfite reductase assigned to five *Desulfobacterota* species could be detected. Functional annotation was based on EggNog v5.0.2, and the assignment of taxonomy was based on Trembl (as of Sept. 2021), and NCBI nr (as of Feb. 2022) database annnotations and manually confirmed against the most recent NCBI nr database via <https://blast.ncbi.nlm.nih.gov/> (blastp, Sept. 2023).

| #pg | Protein function | NSAF | | | Tax_summary |
| --- | --- | --- | --- | --- | --- |
|  |  | Julian Day 107 | Julian Day 128 | Julian Day 144 |  |
| 112 |  | 0 | 0 | 0.00016602 | *Desulfobulbaceae* sp. |
| 182 | Adenylylsulfate reductase | 0 | 0 | 0.00015253 | *Desulfobulbaceae* sp. |
| 293 |  | 5.7524E-05 | 7.7272E-05 | 0 | *Desulfosarcina* sp. |
| 411 | Adenylylsulfate reductase | 0 | 0 | 0.00025035 | *Desulfosarcina* sp. |
| 440 | Dissimilatory sulfite reductase | 0 | 0 | 0.00013727 | *Desulfobacteraceae* sp. |
| 540 |  | 0.00023951 | 0 | 0 | *Desulfobulbaceae* sp. |
| 576 | Dissimilatory sulfite reductase | 0 | 0 | 0.00018468 | *Desulfobulbaceae sp.* |
| 666 |  | 0 | 0 | 0.00015508 | *Desulfosarcina* sp. |
| 743 |  | 5.4294E-05 | 1.8454E-05 | 8.2017E-05 | *Desulfobacterales* sp. |
| 810 |  | 0.0002416 | 0.00015172 | 0.00011497 | *Desulfobacterales* sp. |
| 954 | ATP sulfurylase | 0 | 0 | 0.00016176 | *Desulfobulbaceae* sp. |
| 960 |  | 0 | 0.00017834 | 0.00034704 | *Desulfobacterales* sp. |
| 1214 |  | 0 | 0 | 8.941E-05 | *Desulfobacterales* sp. |
| 1321 |  | 0 | 0.00010197 | 0.00015095 | *Desulfosarcina* sp. |
| 1513 |  | 0 | 0 | 0.00011631 | *Desulfofustis* |
| 2117 |  | 0 | 0 | 0.00026687 | *Desulfobacterales* sp. |
| 2147 | Adenylylsulfate reductase | 0 | 0 | 0.00037822 | *Desulfopila* sp. |
| 2745 |  | 0 | 0 | 0.00026719 | *Desulfofustis* |
| 2868 | Adenylylsulfate reductase | 0 | 0 | 0.00011735 | *Desulfofustis* |
